# Polyneuropathy, Organomegaly, Endocrinopathy, Monoclonal Protein, and Skin Changes (POEMS) Syndrome Presenting With Superior Mesenteric and Portal Vein Thrombosis: A Case Report

**DOI:** 10.7759/cureus.109591

**Published:** 2026-05-25

**Authors:** Chaimae El Aoufir, Sarah Slimani, Omar Ait Sahel, Mohamed Jira, Fadwa Mekouar, Jamal Fatihi

**Affiliations:** 1 Internal Medicine, Mohamed V Military Training Hospital, Rabat, MAR; 2 Neurology, Mohamed V Military Training Hospital, Rabat, MAR; 3 Nuclear Medicine, Mohamed V Military Training Hospital, Rabat, MAR

**Keywords:** case report, monoclonal gammopathy, organomegaly, poems syndrome, polyneuropathy

## Abstract

Polyneuropathy, organomegaly, endocrinopathy, monoclonal protein, and skin changes (POEMS) syndrome is a rare paraneoplastic disorder associated with plasma cell dyscrasia and multisystem involvement. This report describes a case of splanchnic venous thrombosis presenting as the initial clinical manifestation. A 38-year-old woman presented with acute abdominal pain associated with progressive polyneuropathy. Imaging studies revealed extensive thrombosis of the superior mesenteric vein with extension into the portal vein, moderate ascites, hepatosplenomegaly, and diffuse osteosclerotic bone lesions. Laboratory investigations demonstrated an immunoglobulin A (IgA) lambda monoclonal gammopathy. Positron emission tomography-computed tomography showed widespread hypermetabolic osteosclerotic lesions, hypermetabolic axillary lymphadenopathy, and increased cardiac uptake. Transthoracic echocardiography revealed right ventricular dilation with an intermediate probability of pulmonary hypertension. The overall clinical, radiological, and laboratory findings were consistent with disseminated POEMS syndrome. This case illustrates a rare initial presentation of POEMS syndrome manifesting as splanchnic venous thrombosis. It emphasizes the importance of considering POEMS syndrome in patients with unexplained portal hypertension or atypical venous thrombosis in the presence of systemic manifestations.

## Introduction

Polyneuropathy, organomegaly, endocrinopathy, monoclonal protein, and skin changes (POEMS) syndrome is an uncommon multisystem disorder classified among paraneoplastic syndromes and associated with an underlying plasma cell dyscrasia. Its exact incidence remains low, and it is likely underdiagnosed due to its clinical heterogeneity and insidious onset. The syndrome is characterized by a wide spectrum of manifestations, with peripheral neuropathy often representing the most prominent and earliest feature. Other frequently observed findings include hepatosplenomegaly or lymphadenopathy, endocrine abnormalities such as hypothyroidism or hypogonadism, monoclonal gammopathy, and various cutaneous changes.

The pathophysiology of POEMS syndrome is not yet fully understood, but it is believed to involve dysregulated cytokine production, particularly elevated levels of vascular endothelial growth factor (VEGF), which contribute to increased vascular permeability and many of the systemic manifestations. This pro-inflammatory and pro-angiogenic state may also predispose patients to thrombotic events. Indeed, thromboembolic complications have been increasingly recognized in recent years. However, involvement of the splanchnic venous system leading to portal hypertension remains exceptionally rare, especially as an initial presenting feature [1].

We report a case of POEMS syndrome initially presenting with extensive thrombosis of the superior mesenteric vein extending into the portal vein, accompanied by polyneuropathy, cardiac involvement, and diffuse osteosclerotic bone lesions.

## Case presentation

A 38-year-old woman with no prior medical history presented with acute abdominal pain consistent with extensive splanchnic venous thrombosis involving the superior mesenteric and portal veins. Neurological manifestations developed only a few weeks after the onset of abdominal symptoms, initially presenting as progressive bilateral distal paresthesias in the lower limbs with burning and tingling sensations. These symptoms subsequently worsened in a stepwise fashion, evolving into ascending sensorimotor impairment with bilateral proximal and distal lower limb weakness, ultimately confining the patient to bed. Approximately one month later, similar sensory and motor symptoms appeared in the upper limbs. There was no associated bowel or bladder dysfunction. The neurological condition continued to deteriorate progressively over the following months.

On physical examination, the patient was cachectic and exhibited abdominal tenderness and moderate ascites. Neurological examination revealed flaccid areflexic tetraparesis with severely impaired standing and ambulation. Sensory testing demonstrated reduced perception of light touch, pinprick, and vibration, predominantly in the lower limbs. No cutaneous abnormalities or peripheral edema were initially observed.

Initial laboratory investigations revealed microcytic anemia with a hemoglobin level of 8 g/dL and a mean corpuscular volume of 78 fL. Total serum protein was 76 g/L. Liver function tests were within normal limits. Further evaluation for chronic liver disease, including hepatotropic viral serologies and human immunodeficiency virus (HIV) testing, was negative.

Contrast-enhanced abdominal CT demonstrated extensive thrombosis of the superior mesenteric vein extending into the portal vein, moderate ascites, imaging features consistent with portal hypertension, multiple suspicious osteosclerotic lesions, and axillary lymphadenopathy. Upper endoscopy confirmed the presence of esophageal varices, and abdominal ultrasound showed hepatosplenomegaly.

Given the extent and unusual location of the thrombosis, a comprehensive etiological evaluation for prothrombotic conditions was undertaken. Screening for myeloproliferative neoplasms, including testing for the JAK2 V617F mutation, was negative. Evaluation for acquired thrombophilia, particularly antiphospholipid syndrome, including lupus anticoagulant, anticardiolipin, and anti-β2 glycoprotein I antibodies, was also negative. In addition, inherited thrombophilia screening, including Factor V Leiden thrombophilia, Prothrombin G20210A mutation, and protein C, protein S, and antithrombin III levels, revealed no abnormalities.

Laboratory investigations revealed an immunoglobulin A (IgA) lambda monoclonal spike on serum immunofixation, an interleukin-6 (IL-6) level of 25 pg/mL, mildly elevated inflammatory markers, and subclinical hypothyroidism with hypogonadotropic hypogonadism. VEGF levels were pending at the time of diagnostic evaluation and were subsequently found to be >2000 pg/mL.

Electroneuromyography confirmed demyelinating sensorimotor polyneuropathy predominantly affecting the lower limbs. Cerebrospinal fluid analysis revealed albuminocytological dissociation, consistent with POEMS-related inflammatory neuropathy.

Cardiac evaluation showed right axis deviation and incomplete right bundle branch block on electrocardiogram, suggestive of right ventricular strain, consistent with echocardiographic findings of right ventricular dilation and pulmonary hypertension. No low-voltage QRS complexes were observed to suggest cardiac amyloidosis. Given the potential overlap with POEMS syndrome, cardiac magnetic resonance imaging was planned to further assess for myocardial infiltration.

**Figure 1:**
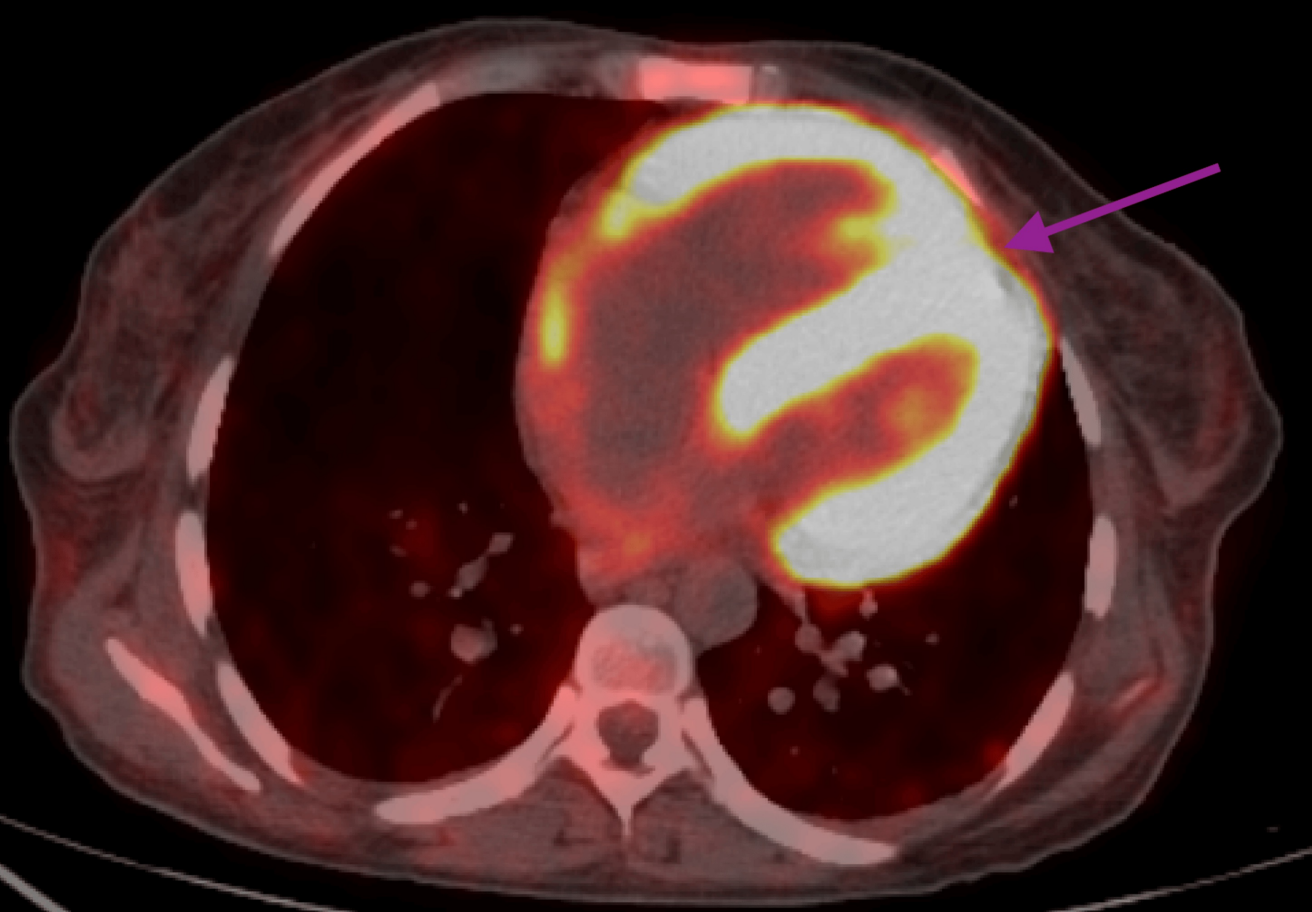
Intense FDG uptake in the cardiac cavities on PET imaging FDG, fluorodeoxyglucose; PET, positron emission tomography

Whole-body positron emission tomography-computed tomography (PET-CT) showed diffuse hypermetabolic osteosclerotic lesions, bilateral hypermetabolic axillary lymphadenopathy, and intense hypermetabolism of the cardiac chambers, predominantly involving the ventricles and interventricular septum, as shown in Figure 1.

The differential diagnosis included disorders within the spectrum of plasma cell dyscrasias. POEMS syndrome may be associated with an underlying osteosclerotic variant of multiple myeloma. Multicentric Castleman disease, a major diagnostic criterion of POEMS syndrome, was considered part of the same disease spectrum. Amyloidosis was also evaluated, given its potential coexistence with plasma cell dyscrasias. Other considerations included systemic inflammatory, myeloproliferative, and hypercoagulable disorders responsible for splanchnic thrombosis. Nevertheless, the coexistence of IgA lambda monoclonal gammopathy, polyneuropathy, diffuse osteosclerosis, organomegaly, lymphadenopathy, elevated cytokine levels, and multiorgan involvement strongly favored the diagnosis of POEMS syndrome.

**Figure 2:**
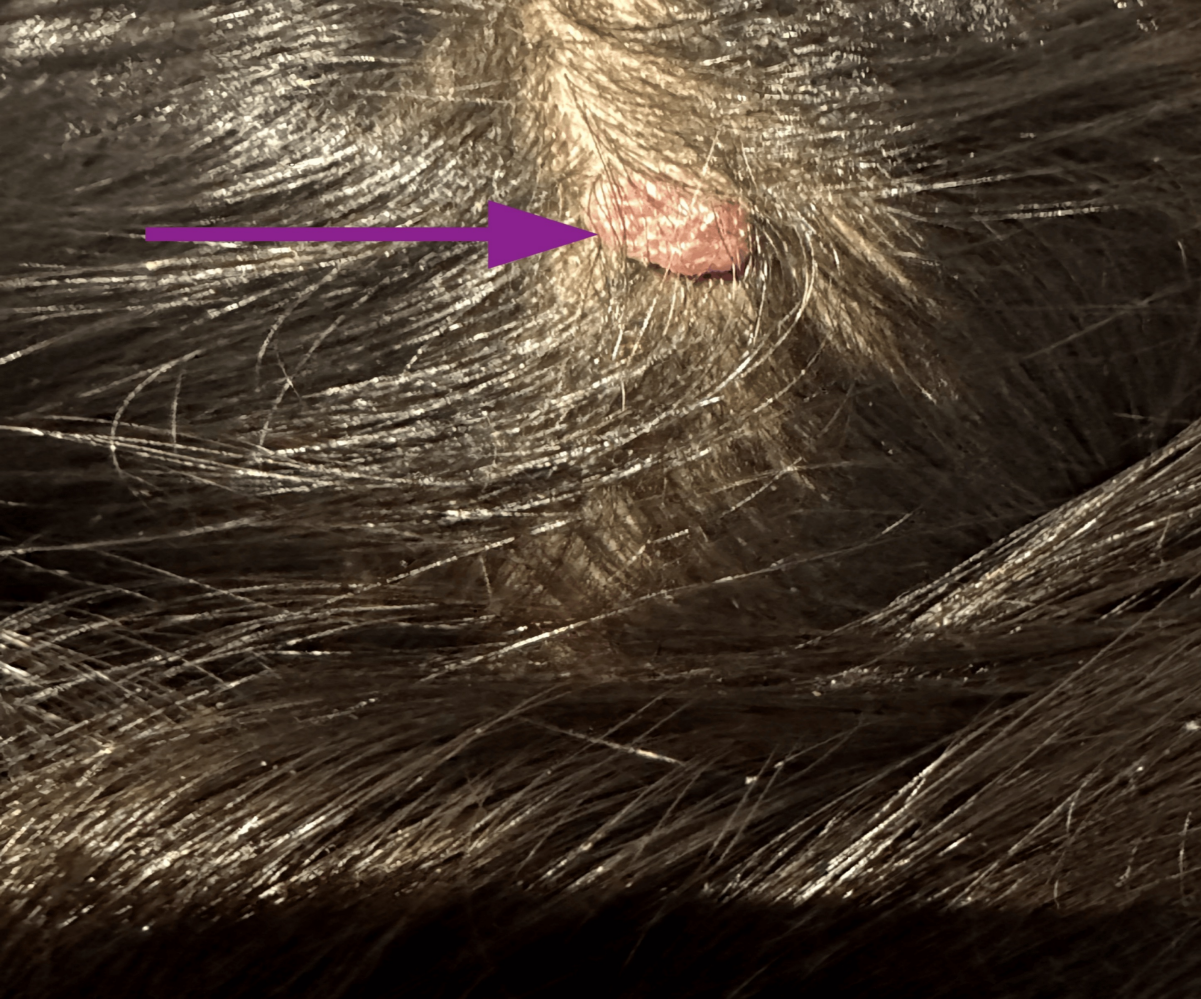
Clinical image of the patient showing cutaneous lesions

**Figure 3:**
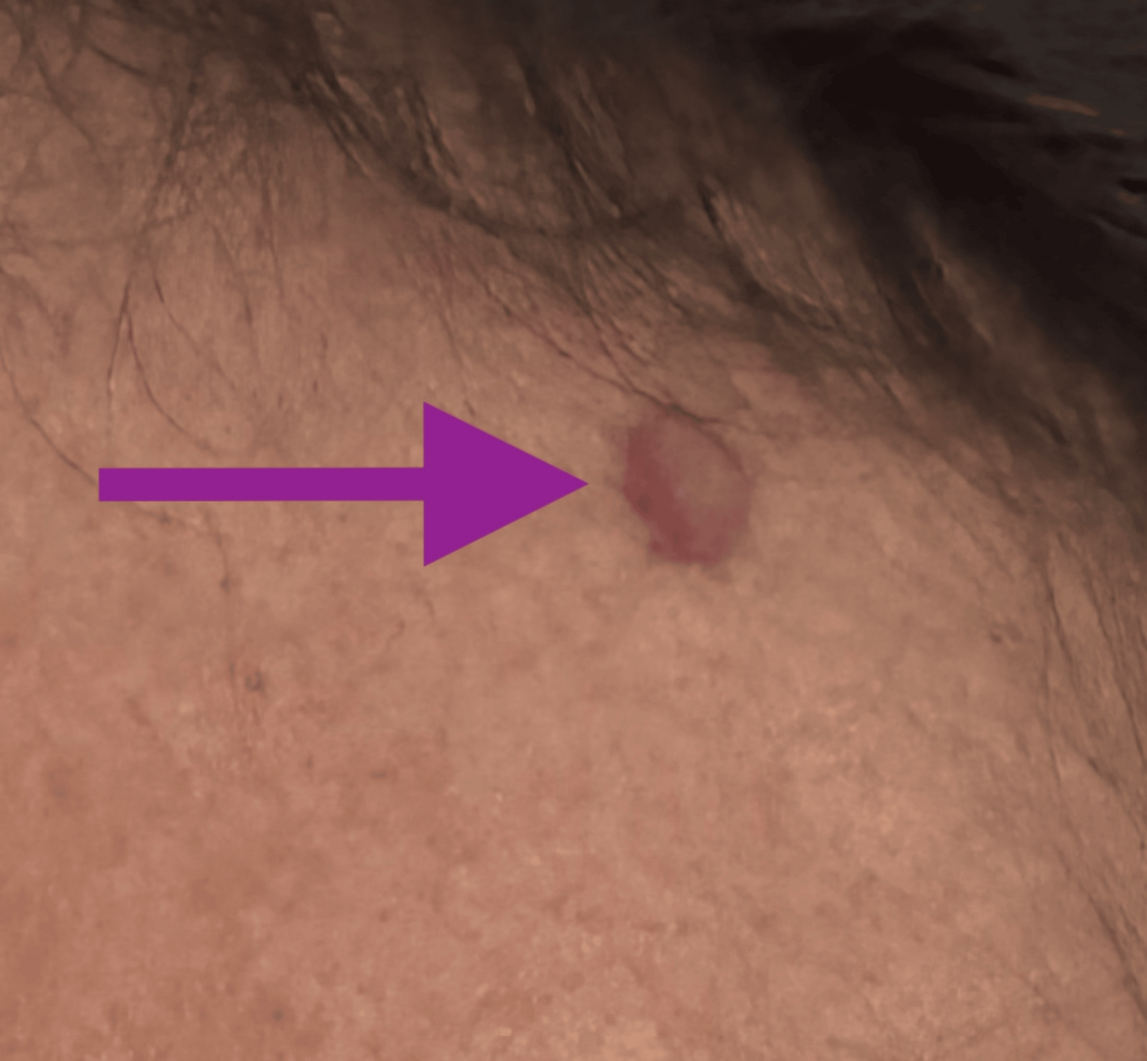
Clinical image of the patient showing another cutaneous lesion

Subsequently, cutaneous examination revealed multiple waxy, firm, yellowish-to-erythematous papules, as illustrated in Figure 2 and Figure 3, clinically suggestive of glomeruloid hemangioma, a characteristic manifestation of POEMS syndrome. Associated findings included diffuse hyperpigmentation, generalized hypertrichosis, and skin thickening, without sclerodactyly. Nail examination was unremarkable, with no leukonychia or Terry’s nails. These findings provided additional support for the diagnosis.

A bone marrow biopsy was performed and demonstrated no evidence of tumor infiltration or features suggestive of multiple myeloma. An excisional biopsy of the axillary lymph node was also undertaken to further evaluate the lymphadenopathy. Histopathological analysis revealed a Castleman-like lymphoproliferative pattern without significant IL-6 overexpression. Overall, these findings were more consistent with POEMS-associated Castleman-like lymphadenopathy than with true multicentric Castleman disease [2] (Table 1).

**Table 1:** Summary of key biological findings VEGF, vascular endothelial growth factor; POEMS, polyneuropathy, organomegaly, endocrinopathy, monoclonal protein, and skin changes; IgA, immunoglobulin A; IL: interleukin; HIV, human immunodeficiency virus

Category	Investigation	Patient result	Clinical interpretation
Hematologic findings	Hemoglobin	8 g/dL	Consistent with anemia
	Mean corpuscular volume	78 fL	Consistent with microcytic anemia
	Inflammatory markers	Mildly elevated	Suggestive of systemic inflammatory activity
Biochemical findings	Total serum protein	76 g/L	Within normal range
	Liver function tests	Within normal limits	No biochemical evidence of hepatic dysfunction
Infectious workup	Hepatotropic viral serologies	Negative	No evidence of chronic viral hepatitis
	HIV serology	Negative	HIV infection excluded
Monoclonal and cytokine studies	Serum immunofixation	IgA lambda monoclonal gammopathy	Supports monoclonal plasma cell disorder
	IL-6	25 pg/mL	Elevated cytokine level
	VEGF	Greater than 2000 pg/mL	Major diagnostic criterion for POEMS syndrome
Endocrine evaluation	Thyroid function tests	Subclinical hypothyroidism	Consistent with endocrinopathy associated with POEMS syndrome
	Gonadal hormonal profile	Hypogonadotropic hypogonadism	Consistent with endocrine involvement
Acquired thrombophilia screening	Lupus anticoagulant	Negative	Antiphospholipid syndrome not supported
	Anticardiolipin antibodies	Negative	Antiphospholipid syndrome not supported
	Anti-β2 glycoprotein I antibodies	Negative	Antiphospholipid syndrome not supported
Inherited thrombophilia screening	Factor V Leiden mutation	Negative	Inherited thrombophilia excluded
	Prothrombin G20210A mutation	Negative	Inherited thrombophilia excluded
	Protein C activity	Normal	Protein C deficiency excluded
	Protein S activity	Normal	Protein S deficiency excluded
	Antithrombin III level	Normal	Antithrombin deficiency excluded
Myeloproliferative neoplasm evaluation	JAK2 V617F mutation	Negative	Myeloproliferative neoplasm not supported
Neurological investigations	Cerebrospinal fluid analysis	Albuminocytologic dissociation	Consistent with inflammatory demyelinating neuropathy
Histopathological findings	Bone marrow biopsy	No tumor infiltration or features of multiple myeloma	Osteosclerotic myeloma not demonstrated
	Axillary lymph node biopsy	Castleman-like lymphoproliferative pattern without significant IL-6 overexpression	Compatible with POEMS-associated Castleman-like lymphadenopathy

POEMS syndrome is defined by a combination of mandatory, major, and minor criteria. Diagnosis requires both mandatory criteria: (1) polyneuropathy and (2) a monoclonal plasma cell disorder (typically lambda-restricted). In addition, at least one major criterion is required, including sclerotic bone lesions, Castleman disease, or elevated VEGF levels. Finally, at least one minor criterion is required, such as organomegaly, extravascular volume overload, endocrinopathy, skin changes, papilledema, or thrombocytosis/polycythemia [3].

In the present case, both mandatory criteria were fulfilled, with a demyelinating polyneuropathy confirmed by electroneuromyography and a monoclonal IgA lambda gammopathy. Multiple major criteria were also present, including diffuse osteosclerotic bone lesions and markedly elevated VEGF levels. Furthermore, several minor criteria were identified, including organomegaly (hepatosplenomegaly and lymphadenopathy), extravascular volume overload (ascites), endocrinopathy (hypothyroidism and hypogonadism), and later skin changes. Altogether, these findings satisfy the diagnostic criteria for POEMS syndrome.

Therapeutic management included anticoagulation with low-molecular-weight heparin for splanchnic venous thrombosis and lenalidomide combined with dexamethasone, consistent with contemporary treatment strategies for disseminated POEMS syndrome. Supportive measures targeted portal hypertension and ascites, nutritional rehabilitation, and monitoring for cardiac and neurological progression. Autologous stem cell transplantation may be considered following clinical stabilization and hematologic response.

Management of the patient’s sensorimotor polyneuropathy consisted of a comprehensive, supportive, and multidisciplinary approach implemented in parallel with disease-modifying therapy. In view of the marked functional disability, a structured neurorehabilitation program was initiated, incorporating passive and active-assisted physiotherapy aimed at preserving joint range of motion, preventing fixed contractures, and progressively improving muscle strength as tolerated. In addition, targeted nutritional support and optimized nursing care were provided to address severe cachexia and global deconditioning, with the objective of enhancing functional reserve and supporting neurological recovery under systemic treatment.

Immunomodulatory strategies such as intravenous immunoglobulin (IVIG) and plasmapheresis were not pursued. This therapeutic decision was based on the pathophysiological mechanisms underlying POEMS syndrome, in which peripheral neuropathy is predominantly mediated by a monoclonal plasma cell dyscrasia with overproduction of pro-angiogenic and pro-inflammatory cytokines, particularly VEGF, rather than a primary antibody-driven immune neuropathy. As a result, plasma exchange and IVIG have shown limited and inconsistent clinical benefit in this setting. Consequently, management was directed toward clonal control using lenalidomide combined with dexamethasone, which targets the underlying plasma cell population and reduces cytokine burden, and therefore constitutes a key disease-modifying therapeutic strategy in POEMS-associated neuropathy.

At the last follow-up, the patient exhibited a favorable clinical course under treatment with lenalidomide and dexamethasone. A progressive improvement in overall functional and nutritional status was observed, with partial reversal of the initial cachectic state. Ascites regressed significantly, consistent with a reduction in extravascular volume overload. Hematologic and inflammatory parameters improved substantially after two treatment cycles, with hemoglobin increasing from 8 g/dL to 11 g/dL and a marked decrease in C-reactive protein levels, alongside a significant reduction in VEGF levels, reflecting biological disease control.

Neurologically, the patient showed a mild but meaningful improvement, characterized by partial recovery of both sensory and motor deficits. This included improved distal strength in the lower limbs and partial restoration of proprioceptive and tactile sensation, allowing better functional mobility with assistance. No new thrombotic events or disease progression were identified during follow-up, supporting an overall positive early therapeutic response.

## Discussion

POEMS syndrome is a rare multisystem disorder driven by an underlying clonal plasma cell dyscrasia, most commonly characterized by a lambda-restricted monoclonal population. Although its exact pathogenesis remains incompletely defined, accumulating evidence supports a central role for cytokine dysregulation, particularly overexpression of VEGF, which is strongly implicated in endothelial dysfunction, increased vascular permeability, and the development of key clinical manifestations such as organomegaly, effusions, and volume overload. IL-6 is also increasingly recognized as an important mediator within this inflammatory milieu and is closely linked to Castleman disease, which may coexist with or mimic POEMS syndrome. In such cases, Castleman-like lymphadenopathy is often considered part of a shared cytokine-driven spectrum rather than a distinct primary lymphoproliferative disorder. Although thrombotic complications have been increasingly reported in POEMS syndrome, the presentation of splanchnic venous thrombosis as the initial clinical manifestation remains exceptionally rare [4,5].

POEMS syndrome belongs to the broader group of plasma cell dyscrasias, which also includes multiple myeloma and AL amyloidosis. These entities represent distinct clinicopathological expressions of clonal plasma cell disorders. Multiple myeloma is defined by malignant plasma cell proliferation with end-organ damage, whereas AL amyloidosis is characterized by tissue deposition of misfolded immunoglobulin light chains leading to progressive organ dysfunction. In contrast, POEMS syndrome is not primarily a tumor-burden or deposition disorder but rather a paraneoplastic cytokine-mediated condition driven by a relatively small but biologically active plasma cell clone.

Castleman disease occupies a relevant position within this spectrum, as it may coexist with POEMS syndrome or present with overlapping clinical and histopathological features. In POEMS-associated Castleman-like lymphadenopathy, lymph node changes are generally interpreted as reactive and cytokine-driven, with IL-6 playing a central role in lymphoproliferation and systemic inflammatory activation. This further supports the concept that POEMS syndrome, Castleman disease, and related manifestations may represent different clinical expressions of a shared cytokine dysregulation axis involving VEGF and IL-6.

The clinical presentation in our patient reflects this underlying biological framework. The occurrence of thrombosis as an initial manifestation may precede the development of more classical features such as neuropathy or skin changes, contributing to diagnostic delay. In this case, elevated cytokines, including IL-6 and markedly increased VEGF, likely contributed to endothelial activation, vascular instability, and a prothrombotic state. The patient subsequently developed a typical pattern of multisystem involvement, including osteosclerotic bone lesions, demyelinating polyneuropathy, lymphadenopathy, portal hypertension with ascites, and cardiac involvement. Associated endocrinopathies, including subclinical hypothyroidism and hypogonadotropic hypogonadism, further reinforced the systemic nature of the disease. Whole-body PET-CT imaging was particularly valuable in defining disease burden and supporting diagnostic orientation.

From a mechanistic perspective, the osteosclerotic lesions observed in POEMS syndrome are considered to result from cytokine-mediated osteoblastic activation driven by the underlying plasma cell clone, in contrast to the lytic lesions typically seen in multiple myeloma. Similarly, the neuropathy in POEMS syndrome is predominantly demyelinating and immune-mediated, likely secondary to cytokine-driven microvascular and endoneurial dysfunction rather than direct tissue infiltration. This differs fundamentally from AL amyloidosis, in which neuropathy results from deposition of amyloid fibrils within peripheral nerves [6,7].

Overall, the coexistence of osteosclerotic bone lesions, demyelinating neuropathy, endocrinopathy, organomegaly, elevated VEGF levels, and systemic inflammatory activation in this patient is highly characteristic of POEMS syndrome and highlights its position within the broader spectrum of plasma cell dyscrasias. This case further emphasizes the overlapping yet distinct biological mechanisms linking POEMS syndrome, Castleman disease, and other monoclonal plasma cell disorders.

The management of POEMS syndrome is evolving and depends on disease extent and transplant eligibility. In localized disease, radiotherapy targeting the dominant plasma cell clone may be sufficient, whereas systemic disease is best treated with autologous stem cell transplantation in eligible patients, which can induce sustained hematologic and neurological improvement.

For patients who are not transplant candidates, immunomodulatory agents such as lenalidomide combined with dexamethasone represent an effective therapeutic option, with documented clinical benefit and reduction in VEGF levels. Proteasome inhibitors may also be considered; however, their use requires caution due to potential neurotoxicity.

More recently, anti-CD38 monoclonal antibodies such as daratumumab have shown promising activity in refractory cases, although available evidence remains limited. Overall, contemporary management strategies increasingly favor a personalized approach targeting both the underlying plasma cell clone and the associated cytokine-driven systemic manifestations [8].

## Conclusions

This case highlights a rare presentation of POEMS syndrome, initially manifesting as extensive mesenteric and portal vein thrombosis with early portal hypertension. Diffuse osteosclerosis, lymphadenopathy, polyneuropathy, endocrinopathy, and cardiac involvement supported a disseminated form of the disease. Clinicians should consider POEMS syndrome in patients with unexplained splanchnic thrombosis accompanied by systemic manifestations.

## References

[1] Talbot A, Jaccard A, Arnulf B (2021). POEMS syndrome: diagnosis, stratification, treatments [Article in French]. Rev Med Interne.

[2] Hmimsa A, Touihem N, Attifi H, Hmidi M (2025). Castleman disease with systemic manifestation: a case report [Article in French]. Pan Afr Med J.

[3] Dispenzieri A (2019). POEMS syndrome: 2019 update on diagnosis, risk-stratification, and management. Am J Hematol.

[4] Dispenzieri A (2023). POEMS syndrome: update on diagnosis, risk-stratification, and management. Am J Hematol.

[5] Brown R, Ginsberg L (2019). POEMS syndrome: clinical update. J Neurol.

[6] Adams D, Lozeron P, Theaudin M, Ribrag V, Bourhis JH, Lacroix C (2011). New elements in the diagnosis and the treatment of primary AL amyloid polyneuropathy and neuropathy due to POEMS syndrome [Article in French]. Rev Neurol (Paris).

[7] Rasch S, Lund T, Asmussen JT, Lerberg Nielsen A, Faebo Larsen R, Østerheden Andersen M, Abildgaard N (2020). Multiple myeloma associated bone disease. Cancers.

[8] Husain A, Simpson RJ Jr, Joodi G (2018). Serum calcium and risk of sudden cardiac arrest in the general population. Mayo Clin Proc.

